# Current Therapy and Therapeutic Targets for Microsporidiosis

**DOI:** 10.3389/fmicb.2022.835390

**Published:** 2022-03-09

**Authors:** Junhong Wei, Zhihui Fei, Guoqing Pan, Louis M. Weiss, Zeyang Zhou

**Affiliations:** ^1^State Key Laboratory of Silkworm Genome Biology, Southwest University, Chongqing, China; ^2^Chongqing Key Laboratory of Microsporidia Infection and Control, Southwest University, Chongqing, China; ^3^Key Laboratory for Sericulture Functional Genomics Biotechnology of Agricultural Ministry, Southwest University, Chongqing, China; ^4^Department of Pathology, Albert Einstein College of Medicine, Bronx, NY, United States; ^5^Department of Medicine, Albert Einstein College of Medicine, Bronx, NY, United States; ^6^College of Life Sciences, Chongqing Normal University, Chongqing, China

**Keywords:** microsporidia, microsporidiosis, therapeutic targets, fumagillin, chitin synthase

## Abstract

Microsporidia are obligate intracellular, spore-forming parasitic fungi which are grouped with the Cryptomycota. They are both opportunistic pathogens in humans and emerging veterinary pathogens. In humans, they cause chronic diarrhea in immune-compromised patients and infection is associated with increased mortality. Besides their role in pébrine in sericulture, which was described in 1865, the prevalence and severity of microsporidiosis in beekeeping and aquaculture has increased markedly in recent decades. Therapy for these pathogens in medicine, veterinary, and agriculture has become a recent focus of attention. Currently, there are only a few commercially available antimicrosporidial drugs. New therapeutic agents are needed for these infections and this is an active area of investigation. In this article we provide a comprehensive summary of the current as well as several promising new agents for the treatment of microsporidiosis including: albendazole, fumagillin, nikkomycin, orlistat, synthetic polyamines, and quinolones. Therapeutic targets which could be utilized for the design of new drugs are also discussed including: tubulin, type 2 methionine aminopeptidase, polyamines, chitin synthases, topoisomerase IV, triosephosphate isomerase, and lipase. We also summarize reports on the utility of complementary and alternative medicine strategies including herbal extracts, propolis, and probiotics. This review should help facilitate drug development for combating microsporidiosis.

## Introduction

Microsporidia are a diverse group of obligate intracellular pathogens phylogenetically related to the Cryptomycota, they form a basal branch on the phylogenic tree of fungi (James et al., 2013). The host range of microsporidia extends from protists to vertebrates (James et al., 2013). These organisms have several adaptations to intracellular life including having mitochondrial remnants termed mitosomes (Williams et al., 2002), small ribosomes with fewer component proteins and rRNA with reduced domains (Peyretaillade et al., 1998), an unstacked type of Golgi apparatus, and a lack of peroxisomes (Beznoussenko et al., 2007; Vavra and Lukes, 2013). All microsporidia have a dormant extracellular spore stage with a limiting spore wall and the spore contains a specialized invasion organelle (the polar tube) and the infective sporoplasm. Upon appropriate environmental stimulation, the polar tube extrudes (germinates) allowing the microsporidia to inject the sporoplasm into host cells and start to proliferate intracellularly (Weidner, 1976).

Horizontal transmission of these infectious spores primarily relies on the fecal-oral route. Another route of transmission is through contact of spores with eye, mucosa, or broken skin. Several microsporidia can also be vertically transmitted to the offspring of infected animals. Depending on the species of microsporidia some can switch hosts across disparate taxa, while other species are relatively host specific (Solter and Maddox, 1998; Andreadis et al., 2012). Symptoms due to infection can vary from asymptomatic carriage to death. Many microsporidia alter host cell biology and infection can result in the development of xenomas, juvenilization of the host, and metabolic changes in infected cells. Microsporidia have been classified by the National Institutes of Allergy and Infectious Diseases (NIAID) and the Centers for Disease Control and Prevention (CDC) as Category B biodefense priority pathogens.^1^

Microsporidiosis is usually self-limiting or asymptomatic in the general human population, but more severe in immune deficient patients. The importance of microsporidia in humans came into focus with the emergence of the acquired immune deficiency syndrome (AIDS). In humans severe infection has been seen in patients with AIDS, patients following organ transplantation, and in patients taking immune suppressive drugs or immune modulatory antibodies (Han and Weiss, 2017; Dumond et al., 2021; Dumortier et al., 2021). Most commonly infection results in a diarrheal syndrome; however, patients may also suffer from encephalitis, ocular infection, sinusitis, myositis, or disseminated infection (Weber et al., 1994; Weiss and Keohane, 2010; Ziad et al., 2021). Among the approximately 1,500 microsporidian species, 17 species can infect humans. *Enterocytozoon bieneusi* is the most frequently reported species causing gastrointestinal disease in immune-deficient individuals, followed by *Encephalitozoon intestinalis* (Garcia-Torres et al., 2018; Li et al., 2019). Less common human-infecting microsporidian species include *Anncaliia*, *Vittaforma*, *Trachipleistophora*, and *Pleistophora* (Shadduck et al., 1990; Field et al., 1996; Cali and Takvorian, 2003; Cali et al., 2010; Boileau et al., 2016; Anderson et al., 2019). Livestock animals are the most frequently identified reservoir hosts, transmission can occur from livestock excreta and wastewater that contain spores or *via* spore-contaminated livestock products (Li et al., 2020; Ruan et al., 2021). Some zoonotic microsporidia transmission cases have been caused by infections in companion animals, such as cats and dogs, which may become potential public health risks (Cama et al., 2007; Karim et al., 2014; Wan et al., 2016; Ruan et al., 2021). Wild animals are also potential pathogen reservoirs, like monkeys, foxes, raccoons, beavers, and dolphins (Sulaiman et al., 2003; Desoubeaux et al., 2018; Zhao et al., 2021). Several outbreaks of microsporidiosis in healthy individuals have also been reported (Kwok et al., 2013; Lam et al., 2013; Wang et al., 2018). It is likely that microsporidiosis is a common infection but is self-limited or asymptomatic in healthy hosts. To this end, microsporidia have been identified in up to 20% of children with diarrhea in underdeveloped countries (Gumbo et al., 1999).

Microsporidiosis caused by *Nosema bombycis* in silkworm, i.e., pébrine, is one of the most deadly diseases seen in the sericulture industry. Transmission of this infection can occur both horizontally and vertically, causing heavy losses or even total crop failure. Historically pébrine caused a collapse of the French and Italian silk industry in the mid-19th century, until Pasture was able to develop preventative methods and save the silkworm industry (Bhat et al., 2009; Hukuhara, 2011). This disease continues to be an important concern for silkworm growers and scientists in sericulture practicing countries around the world and remains a major threat to sericulture.

Honey bees are key pollinators of both wild plant communities and agricultural crops, they are important to the environment as well as the food supply (Calderone, 2012). *Nosema ceranae* and *Nosema apis* are major causes of microsporidiosis in honey bees (Higes et al., 2008; Fries, 2010). *N. ceranae* is now the predominant microsporidium species seen in the western honey bee (*Apis mellifera*), which is the most important bee species for honey production and animal-mediated pollination (Williams et al., 2014). While the antimicrosporidian drug fumagillin continues to be effective against *N. apis*, it has not been as effective for infections due to *N. ceranae* (Williams et al., 2008). *N. apis* and *N. ceranae* have recently been redefined as *Vairimorpha apis* and *Vairimorpha ceranae* based on a molecular phylogenetics analysis of the Nosema and Vairimorpha clades (Tokarev et al., 2020). For the purposes of this review the *Nosema* Genus will still be used.

Shrimp aquaculture is an important long-term sustainable industry for many developing countries (Flegel, 1997). Microsporidiosis is the most common and harmful eukaryotic pathogen to shrimp, and directly threatens shrimp aquaculture (Morado, 2011; Tang et al., 2015). *Enterocytozoon hepatopenaei* (EHP) and *Agmasoma penaei* are well-known species which cause economic losses in shrimp aquaculture. *A. penaei* can infect muscle and connective tissues of giant tiger shrimp *Penaeus monodon* and pacific white shrimp *Litopenaeus vannamei.* Infected shrimp are called “cotton shrimp” or “milk shrimp” due to the whitish or milky appearance seen on various parts of the body (Pasharawipas and Flegel, 1994; Prasertsri et al., 2009). Infected shrimp are not able to be sold leading to economic losses. EHP infects the hepatopancreas of shrimp damaging the functions of this critical organ and causing slow or stunted growth in infected shrimp (Thitamadee et al., 2016). In addition, EHP infection increase the susceptibility of shrimp to acute hepatopancreatic necrosis disease caused by *Vibrio* (Aranguren et al., 2017). EHP has been widely found in Asia and other parts of the world, severely impacting aquaculture production.

Various types of medicinal regimes have been used to treat microsporidiosis, the results of therapy have ranged from disappointing to promising. Previous reviews have summarized the drugs and therapeutic targets for the treatment of microsporidiosis in humans (Conteas et al., 2000; Han and Weiss, 2018). The objective of this review is to summarize recent studies on new drugs and targets for the treatment of these infections in both vertebrates and invertebrates.

## Current Microsporidian Therapies and Their Targets

As microsporidia were once considered “primitive” protozoa, many anti-protozoan drugs have been tested as therapeutic agents. The majority of these anti-protozoan drugs have not been effective for the treatment of microsporidiosis. The widely used anti-protozoan and anti-bacterial agent trimethoprim-sulfamethoxazole has minimal activity against either *Ent. bieneusi* or *Enc. intestinalis* (Conteas et al., 2000). While metronidazole was shown *in vitro* to inhibit the germination of *Enc. intestinalis*, clinical studies demonstrated it was ineffective against microsporidiosis (Canning and Hollister, 1990; Orenstein, 1991; He et al., 1996). Studies on the effectiveness of paromomycin have also been disappointing, and sequence data has shown that microsporidia lack the rRNA binding site for this drug (Beauvais et al., 1994). The target of many antifungal drugs is the ergosterol biosynthesis pathway, however microsporidia lack this typical fungal pathway, thus amphotericin B has not been effective (Beauvais et al., 1994; Albrecht et al., 1995). Atovaquone has also been ineffective for therapy, consistent with the absence of mitochondria in these pathogens. Itraconazole has demonstrated some microsporidian growth inhibition *in vitro*; however it has not been effective as a solitary agent in clinical microsporidiosis. Itraconazole has been used in combination with other drugs (e.g., fumagillin or albendazole) in several clinical cases. Current recommended therapeutic options, which directly inhibit microsporidian infections are quite limited, include only two structural classes of drugs: benzimidazoles and terpenes.

**FIGURE 1:**
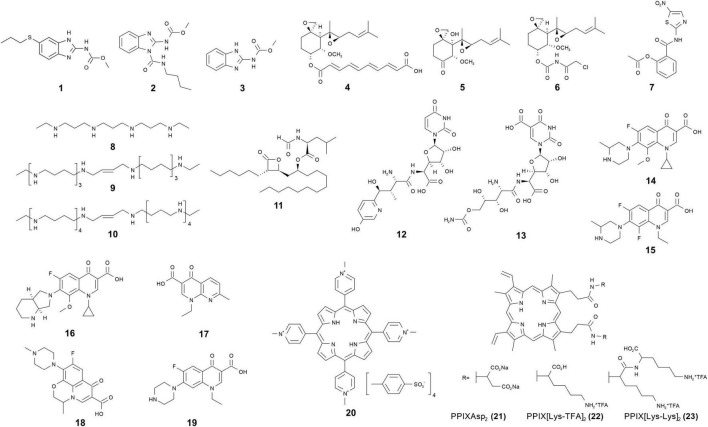
∣ Chemical structures of antimicrosporidial agents.

Benzimidazole compounds share a bicyclic compound consisting of the fusion of benzene and imidazole (Figure 1). They exhibit various types of bioactivity, including anti-inflammatory, anti-hypertensive, anti-bacterial, anti-parasitic, and anti-fungal effects (Labanauskas et al., 2004; Navarrete-Vazquez et al., 2010; Hosamani and Shingalapur, 2011). Benzimidazoles are well-known therapeutic agents with broad-spectrum anthelmintic ability and also activity against fungi (in commercial applications where they inhibit mold growth), they act mainly through inhibition of the microtubule assembly (Lacey, 1990). Albendazole **(1)** has a broad spectrum of activity against helminths with minimal host side effects. It has been licensed for human use in various parts of the world (Horton, 2000). Albendazole has been shown to control microsporidiosis caused by *Encephalitozoon* spp. and the β-tubulin sequences of these Encephalitozoonidae confirms the presence of amino acid residues associated with sensitivity to benzimidazoles (Li et al., 1996; Didier, 1997). There are numerous case reports demonstrating the efficacy of albendazole for microsporidiosis caused by *Encephalitozoon* spp., including diarrhea caused by *Enc. intestinalis*, chronic disseminated infection caused by *Enc. hellem* and disseminated infection caused by *Enc. cuniculi* involving the central nervous system (Lecuit et al., 1994; Dore et al., 1995; Weber et al., 1997). In immunocompetent children with diarrhea caused by microsporidium, albendazole therapy seems effective in reducing microsporidial excretion in feces (Tremoulet et al., 2004). Albendazole is also effective against myositis caused by *Trachipleistophora hominis*, *Anncaliia algerae*, and *Anncaliia vesicularum* (Field et al., 1996; Cali et al., 1998; Watts et al., 2014; Sutrave et al., 2018). An *in vitro* assay demonstrated that albendazole was less effective against *Vittaforma corneae* than against *Enc. intestinalis* or *Enc. cuniculi* (Didier, 1997; Franzen and Salzberger, 2008). Studies of patients with diarrhea caused by *Ent. bieneusi* who were treated with albendazole proved that the effect of albendazole is quite limited (Dieterich et al., 1994; Leder et al., 1998). This is consistent with the presence of amino acids associated with albendazole resistance in the β-tubulins sequences of *Enterocytozoon* and *Vittaforma* (Akiyoshi et al., 2007; Franzen and Salzberger, 2008). There are also some albendazole resistance variations in different genotypes of *Encephalitozoon* (Kotkova et al., 2017). Some research suggests that *Enc. cuniculi* genotype III show elevated resistance to albendazole treatment in immune deficient and immune competent mice (Sak et al., 2020). Based on NIH’s guidelines, albendazole is recommended for treatment of intestinal and disseminated microsporidiosis caused by microsporidia other than *Enc. bieneusi* and *V. corneae*.

Benomyl **(2)** and carbendazim **(3)** are another type of benzimidazoles that are wildly used as fungicide for fungal diseases in field crops (Figure 1). They are also considered as medicinal regimes for microsporidiosis in insects. Many studies suggest that benomyl and its derivative compounds are effective in controlling microsporidiosis in insects such as *Nosema heliothidis* in *Heliothis zea* and *Nosema kingi* in *Drosophila willistoni* (Armstrong, 1976; Brooks et al., 1978). Ultrastructural evidence suggested that carbendazim causes elongation, vacuolation, and depletion of cytoplasmic contents of the of *N. bombycis* in meront and spore stages (Jyothi et al., 2005). In China, carbendazim formulations have been registered and commercially marketed to control *N. bombycis* in silkworm seed production. However, due to its hepatotoxicity and toxic effects in animal reproduction (Barnes et al., 1983; Sakr et al., 2004), benomyl and carbendazim have not been used for the treatment of mammalian microsporidiosis.

Fumagillin **(4)**, ovalicin **(5)**, and their analogs are terpenes share unique structures with a cyclohexane framework, two epoxides and three or four contiguous stereogenic centers situated on the cyclohexane ring (Figure 1). Originally isolated from the fungus *Aspergillus fumigatus*, fumagillin was initially utilized as a treatment for amebiasis (McCowen et al., 1951; Killough et al., 1952). As more effective drugs for amebiasis were developed, it was abandoned. Researchers subsequently discovered that fumagillin had both antimicrosporidial and anti-angiogenesis activities (Lefkove et al., 2007). Fumagillin selectively and covalently binds to methionine aminopeptidase type 2 (MetAP2), however, it does not inhibit methionine aminopeptidase type 1 (MetAP1) (Sin et al., 1997). As removal of an N-terminal methionine is often essential for protein function and post-translational modification, inhibition of methionine aminopeptidase can affect eukaryotic cell survival. Mammalian cells possess both MetAP1 and MetAP2 while genome data from *Enc. cuniculi* and other microsporidian indicate that microsporidia lack MetAP1; hence fumagillin could selectively inhibit the growth of microsporidia in mammalian cells. MetAP1 can compensate for the inhibition of MetAP2 by fumagillin for the eukaryotic host cells, but inhibition of MetAP2 in microsporidia inhibits an essential enzyme in the pathogens. Unlike albendazole, fumagillin has been shown to be effective against *Ent. bieneusi*. Intestinal infection patients with dosages of 60 mg/day exhibited completely clearing of *Ent. bieneusi* (Molina et al., 2000; Molina et al., 2002). In a pediatric case of digestive microsporidiosis in a liver-kidney transplant child, treatment with fumagillin alone successfully eliminated the parasite (Desoubeaux et al., 2013). Fumagillin and a semisynthetic analog TNP-470 **(6)** are also effective against *Enc. cuniculi*, *Enc. hellem*, *Enc. intestinalis*, and *V. corneae* (Diesenhouse et al., 1993; Didier, 1997; Coyle et al., 1998). In a case of disseminated *A. algerae* infection, after failure of albendazole-based therapy, combined use of albendazole and fumagillin treatment resulted in clinical response and patient survival (Boileau et al., 2016). Oral treatment with fumagillin or TNP470 was also shown to control *Loma salmonae* or *Nucleospora salmonis* infection in the Chinook salmon (*Oncorhynchus tshawytscha*) (Higgins et al., 1998). Fumagilin-B soluble powder, a water soluble preparation containing bicyclohexylammonium fumagillin, is widely used to control *N. apis* in infected honeybees around the world. There are concerns about the toxicity of fumagillin and, consequentially, many countries outside of the Americas (including the European Union) have banned fumagillin for agricultural use (European Commission, 2010). In humans, treated for microsporidiosis, fumagillin has caused reversible thrombocytopenia as well as aseptic meningitis (Molina et al., 1997; Audemard et al., 2012).

Nitazoxanide **(7)** was developed and commercialized as an antiprotozoal drug against a broad range of parasites including protozoa, nematodes, cestodes, and trematodes (Rossignol and Maisonneuve, 1984; Cabello et al., 1997; Doumbo et al., 1997). Subsequently, it was also identified as a broad-spectrum antiviral drug, e.g., clinical trials found that oral administration of nitazoxanide reduced the duration of clinical symptoms caused by influenza (Haffizulla et al., 2014). An *in vitro* assay of nitazoxanide demonstrated that it had moderate activity in inhibiting the growth of *Enc. intestinalis* and *V. corneae* (Didier et al., 1998). Two case reports demonstrated that nitazoxanide therapy treatment successfully inhibited microsporidiosis in patients (Bicart-See et al., 2000; Saffo and Mirza, 2019). While the mechanism of action of nitazoxanide is not fully known, the most accepted nitazoxanide target is pyruvate: ferredoxin; however, microsporidia lack pyruvate:ferredoxin oxidoreductase and use pyruvate dehydrogenase in its place (Keeling, 2001; Hoffman et al., 2007). Nitazoxanide also has other targets and a possible nitazoxanide target in microsporidia is protein disulfide isomerase (Muller et al., 2008). In general, more trials are required to evaluate the possible role of nitazoxanide in treating microsporidiosis.

## Experimental Antimicrosporidial Compounds and Their Therapeutic Targets

Commonly known as putrescine, spermidine and spermine, polyamines are low molecular weight organic chemicals with more than two amino groups. Polyamines play an important role in cell growth and differentiation by binding to nucleic acids through electrostatic forces and hydrogen bonding interactions. Due to their important roles in cellular physiology the intracellular concentrations of polyamines are strictly controlled through uptake, synthesis, interconversion, and degradation (Feuerstein et al., 1991; Casero, and Pegg, 1993; Marton and Pegg, 1995; Bacchi et al., 2003). Polyamine concentrations are elevated in tumors compared to normal tissue and, therefore, many researchers have focused on the development of polyamine analogs as anti-cancer therapeutic agents (Hayes et al., 2014). *Enc. cuniculi* has polyamine pathways that include synthesis and back-conversion (Bacchi et al., 2001; Ma et al., 2020). Isotope label studies indicate that the intracellular polyamine levels of pre-emergent *Enc. cuniculi* spores are dependent on uptake and interconversion rather than synthesis (Yarlett and Bacchi, 1988), indicating that synthetic polyamines targeting interconversion enzymes like spermidine spermine, N-acetyl transferase and polyamine oxidase are potential therapeutic agents for microsporidiosis. N′, N″-bis (ethyl) norspermine (BE-3-3-3) **(8)**, a polyamine analogue that induces spermidine spermine N-acetyl transferase and down-regulates polyamine metabolism, inhibited *Enc. cuniculi* growth in RK-13 cells (Coyle et al., 1996; Figure 1). *In vivo* therapeutic studies in immunosuppressed mice demonstrated that synthetic polyamine analogs SL-11144 **(9)** and SL-11158 **(10)** are able to cure microsporidiosis due to *Enc. cuniculi* (Bacchi et al., 2002; Figure 1). In a SCID mouse model, the synthetic polyamine analogues were shown to have superior efficiency against *Ent. bieneusi* to that of fumagillin (Feng et al., 2009). Due to the reliance on uptake rather than synthesis of polyamines, screening, or developing new polyamine analogs appears to be a useful future direction for the development of new therapeutic agents for the treatment of microsporidiosis.

Lipids are vital metabolites for multiplication and differentiation in eukaryotes. The formation of membranous structures is highly reliant on the amount of available lipids. Orlistat **(11)** is a derivative of lipstatin (isolated from *Streptomyces toxytricini*) which can irreversibly and efficiently block pancreatic and gastric lipase (Figure 1). It is minimally absorbed when given orally and its effect is due to inhibition of lipases in the gastrointestinal tract. Orlistat was approved in 1999 by the FDA (United States) for obesity management (Derosa and Maffioli, 2012). Interestingly, orlistat has also been shown to have activity against *Giardia intestinalis (duodenalis*), *Trypanosoma brucei*, and *Mycobacterium tuberculosis* (Parker et al., 2009; Yang et al., 2012; Hahn et al., 2013). In mice infected with either *Ent. bieneusi* or *Enc. intestinalis*, orlistat treatment was shown to decrease the amount of spores seen in stool and the intestinal spore load (Abou-El-Naga et al., 2019). The effect seen was similar to that observed with fumagillin or albendazole treatment. Orlistat may affect these pathogens directly by inhibiting their lipid metabolizing enzymes or indirectly by affecting the lipid supply through inhibition of host enzymes (McClendon et al., 2009; Hahn et al., 2013). Genome analysis of microsporidia indicates that microsporidia lack many genes required for the biosynthesis of many important metabolites including lipids (Nakjang et al., 2013), to this end the antagonistic effect of orlistat on growth may be due to inhibition of the breakdown of lipids by the host, limiting the available lipid precursors for uptake by the microsporidia in the gut. Microsporidia have been shown to have an intact phospholipid metabolic pathway for synthesizing membrane phospholipids and this pathway is different from that of other eukaryotes (i.e., host cells). As phospholipids account for 60% of the total lipids in microsporidia and phosphatidic acid has been proved to be a limiting host metabolite for the proliferation of *Tubulinosema ratisbonensis* in *Drosophila* (El Alaoui et al., 2001a,b; Franchet et al., 2019), their phospholipid biosynthesis pathway may also be a promising therapeutic target.

Chitin is a carbohydrate polymer which provides high resistance to the environment and confers structural rigidity to the spore wall of microsporidia. Chitin is synthesized by a large family of chitin synthase enzymes which can be clustered into seven discernable classes (Roncero, 2002). As chitin is not found in mammalian cells and is an essential component of the microsporidian spores, chitin synthase is a promising target for antimicrosporidial drug development. Nikkomycin and polyoxin are peptide-nucleoside antibiotic which block chitin synthesis and fungal growth (Figure 1). They have structural similarity to UDP-N-acetylglucosamine, a ubiquitous and essential metabolite for chitin synthesis, which allows them to competitively inhibit chitin synthases that use UDP-N-acetylglucosamine to synthesize linear chitin molecules (Groll et al., 1998). Nikkomycin Z **(12)** inhibited the growth of *Enc. hellem* in fetal bovine lung fibroblast cells and the infectivity of any spores that developed in drug-treated cultures (Bigliardi et al., 2000). Polyoxin D **(13)** and nikkomycin Z were shown to reduce the number of parasitic foci in *Enc. cuniculi* infected monkey kidney cells (Sobottka et al., 2002). The non-specific chitin synthase inhibitor lufenuron was shown to inhibit the *in vitro* growth of *Enc. intestinalis* and *V. corneae* (Didier et al., 1998). Fungi have different classes of chitin synthases with distinct functions. For example, *Saccharomyces cerevisiae* has three chitin synthases with distinct functions in cell wall expansion, septum formation, and budding, while filamentous fungi generally have seven or eight distinct chitin synthases (Brosson et al., 2005). This property hindered the application of existing chitin synthase inhibitors, such as polyoxin or nikkomycin, because they usually inhibit one class of chitin synthases, but show less efficiency against other classes of these enzymes. In contrast, microsporidia possess a single type of class IV chitin synthase (Brosson et al., 2005). Drugs that are specifically targeting this type of chitin synthase may be sufficient to inhibit the growth of microsporidia. Microsporidia are obligate intracellular pathogens, unlike many fungi, and thus transportation across the plasma membrane may be the major factor influencing the effectiveness of hydrophilic compounds like nikkomycin and polyoxin.

Quinolones contain a bicyclic core structure related to the compound 4-quinolone. These drugs target type II topoisomerases, DNA gyrases, and type IV DNA topoisomerases of pathogens. They are broad-spectrum synthetic antibiotics which are widely used for the treatment of bacterial infections (Hooper, 2000; Andersson and MacGowan, 2003; Liu and Mulholland, 2005). Fluoroquinolones were derived from quinolones by modifying their structure with fluorine and have the same mechanism of action as quinolones. Genome sequence data demonstrates that *V. corneae* has a gene with high level of identity with DNA topoisomerase IV C subunit, an enzyme previously identified only from prokaryotes (Mittleider et al., 2002). Therefore, researchers have examined the efficacy of (fluoro) quinolones for inhibition of the growth of *V. corneae* (Didier et al., 2005; Figure 1). Their results indicate that gatifloxacin **(14)**, lomefloxacin **(15)**, moxifloxacin **(16)**, and nalidixic acid **(17)** could inhibit *V. corneae* growth *in vitro* and *in vivo*, and that gatifloxacin, lomefloxacin, norfloxacin **(18)**, and ofloxacin **(19)** prolonged survival of *V. corneae*-infected mice. Interestingly, gatifloxacin, lomefloxacin, moxifloxacin, and nalidixic acid could also inhibit *Enc. intestinalis* which does not have an identified type IV topoisomerase in its genome, indicating that quinolones may interact with other targets in addition to classic type IV topoisomerases.

Porphyrins are ubiquitous aromatic heterocyclic compounds in nature, they participate in many important biochemical processes in living organisms, such as oxygen transport and photosynthesis. Traditionally used against cancer, some porphyrins show inhibitory effect against bacteria, viruses, fungi, and protozoa (Jori et al., 2006; Allison and Moghissi, 2013). The synthetic amphiphilic protoporphyrin derivatives TMePyP **(20)** and PPIX(Asp)_2_ **(21)** prevented *N. ceranae* spore development in *A. mellifera* (Ptaszynska et al., 2018; Figure 1). Protoporphyrin derivatives with other amino acid moieties, PPIX[Lys-TFA]_2_ **(22)** and PPIX[Lys-Lys]_2_ **(23)**, were also shown to reduce spore loads in infected honey bees (Buczek et al., 2020). In general, in response to light (or radiation) porphyrins react with oxygen to produce cytotoxic reactive oxygen species such as superoxide, hydrogen peroxide, or hydroxyl radicals, and these highly reactive radicals react with organic substrates to produce cytotoxicity (Castano et al., 2004). However, the mechanism of inactivation of microsporidia is currently not known, as light or radiation does not appear to be involved in the ability of porphyrins to inhibit microsporidia.

## Other Promising Therapeutic Targets

The mechanism by which microsporidia invade host cells is unique. Under appropriate environmental conditions a spore germinates shooting out its polar tube and transferring its sporoplasm into a host cell through the extruded polar tube (Han et al., 2017; Jaroenlak et al., 2020). This is a critical process in the life cycle of these organisms and enzymes or factors involved in spore germination are potential therapeutic drug targets. The exact mechanism of spore germination and polar tube discharge is not well-understood, and conditions that activate spores vary widely among species, including pH condition, cations, and calcium. Experimental evidence using *Glugea hertwigi* has suggested that calcium flux plays an important role in the initiation of spore discharge (Weidner and Byrd, 1982). Displacement of calcium from the polaroplast membrane is thought to trigger polaroplast contraction or combine with the polaroplast matrix to cause polaroplast swelling providing the propulsive force for germination (Weidner, 1982; Weidner and Byrd, 1982). Both calcium antagonists (verapamil and lanthanum) and calmodulin inhibitors (trifluroperazine and chlorpromazine) have been demonstrated to prevent spore germination in *Spraguea lophii* (Pleshinger and Weidner, 1985). The calcium channel blocker nifedipine inhibited the germination of *Enc. hellem* and *Enc. intestinalis* spores *in vitro* (Leitch et al., 1993; He et al., 1996). However, no *in vivo* data exists regarding the use of calcium channel blockers in microsporidiosis.

Based on the osmotic pressure theory of spore germination, water flow across the spore wall and plasma membrane which is accompanied by swelling of the polaroplasts and posterior vacuole after germination, results in quickly increased osmotic pressure and the corresponding eversion of the polar tube and subsequent expulsion of the sporoplasm (Frixione et al., 1997). To this end, aquaporins are also a potential target for inhibiting spore germination, because they are critical proteins for water flow across membranes (Ghosh et al., 2006a,b). The aquaporin inhibitor HgCl_2_ has been shown to be effective in inhibiting *A. algerae* spore germination (Frixione et al., 1997). However, HgCl_2_ is highly toxic and has significant health risks, therefore, while a proof of principle for aquaporin inhibition as a target for inhibition of germination, it is not a useful drug for the treatment of microsporidiosis.

Triosephosphate isomerase is a ubiquitous enzyme which catalyzes the interconversion between triose phosphate isomers dihydroxyacetone phosphate and D-glyceraldehyde-3-phosphate. As the process triosephosphate isomerase catalyzes is essential for the glycolytic pathway and many parasites require this enzyme for efficient energy production, several triosephosphate isomerase inhibitors have been studied in various parasitic infections. A selective inhibitor of *Trypanosoma cruzi* triosephosphate isomerase dramatically reduced parasites in the blood of experimentally infected mice and greatly enhanced their survival rate (Aguilera et al., 2016). Selective *Fasciola hepatica* triosephosphate isomerase inactivators could kill the juvenile form of *F. hepatica* in low concentration and showed low host toxicity (Ferraro et al., 2020). In addition to its effect on energy production, inhibition of triosephosphate isomerase results in accumulation of dihydroxyacetone phosphate or D-glyceraldehyde 3-phosphate, which can be cytotoxic (Han and Weiss, 2018). It has been shown that thiol-reactive compounds like sulbutiamine, rabeprazole, and omeprazole can effectively inhibit the triosephosphate isomerase of *Enc. intestinalis* (Garcia-Torres et al., 2018). As these drugs do not significantly inactivate human triosephosphate isomerase (Garcia-Torres et al., 2016), they may be considered as new potential drugs for treating microsporidiosis.

Analysis of the available sequenced microsporidian genomes on MicrosporidiaDB.org suggests that they have obtained several genes *via* horizontal gene transfer events, such as the ADP/ATP translocase gene family transferred from Chlamydiae and glutamate-ammonia ligase from an unknown prokaryotic source (Pombert et al., 2012). Thymidine kinase is a ley enzyme in the nucleic acid salvage pathway, it catalyzes the phosphorylation of thymidine to thymidine monophosphate. Thymidine kinase is thought to have been lost in the fungal lineage shortly after it diverged from animals (Alexander et al., 2014). However, two independent horizontal transfer events of thymidine kinase have occurred in microsporidia, including transfers of bacterial thymidine kinase genes into several microsporidia taxa and transfer of a putative viral-like thymidine kinase into *Nematocida parisii* (Alexander et al., 2016). Several prodrugs specifically target thymidine kinase, like 5-fluoro-2-deoxyuridine and acyclovir, and these are widely applied to treat simplex virus and several types of cancer cells (Furman and Barry, 1988; Simmons, 2002). Heterologous expression of a microsporidian thymidine kinase in *S. cerevisiae* can convert 5-fluoro-2-deoxyuridine into fluorodeoxyuridine monophosphate, completely inhibited the growth of the transgenic *S. cerevisiae* (Alexander et al., 2016). It is reasonable to postulate that the microsporidia that have thymidine kinase in their genomes maybe susceptible to such thymidine kinase prodrugs.

## Alternative Medicine for Treatment of Microsporidiosis

Most complementary and alternative medicine strategies for the treatment of microsporidiosis focus on the treatment of nosemosis in honey bees. This is because while fumagillin is the only veterinary regiment recommended by the World Organization for Animal Health for treating nosemosis in honey bees it is no longer licensed in the European Union. As a result, there is a need for alternatives to control this disease and several “natural” products have been sold as veterinary treatments for nosemosis such as Nosestat^®^ and Vitafeed Gold^®^. Many of these alternative strategies have now been proven by controlled studies to be ineffective (Botias et al., 2013). More research is clearly needed to develop reliable and effective alternative strategies for the treatment of nosemosis in bees. Experience with these alternative strategies for treating nosemosis in bees should also provide new therapeutic ideas for the treatment of microsporidiosis in human or other animals.

Various plants have been used in traditional herbal remedies around the world and are reported to have numerous pharmacological activities, however, the mechanisms underlying these reported effects and the active substance in the various plant extracts is often unknown. Historically, plants have been sources of pharmacologically active compounds against many types of pathogens and plant extracts have been evaluated and used for the treatment of infectious diseases caused by bacteria, fungi, protozoa, and viruses (Ali-Shtayeh and Abu Ghdeib, 1999; Yoshida et al., 2005; Thembo et al., 2010). Such studies have resulted in new therapeutic agents such as the use of artemisinin, an antimalarial compound derived from *Artemisia carvifolia*. Various plant extracts have been evaluated as therapeutic agents for microsporidiosis. Ethanolic extracts of *Laurus nobilis* were demonstrated to inhibit *N. ceranae* development in *A. mellifera* at a concentration of 1% in syrup (Porrini et al., 2011). Decoction extracts from the Chinese herb *Andrographis paniculata* significantly inhibited proliferation of *N. ceranae* and improved the survival rate of infected bees in a dose-dependent manner (Chen et al., 2021). In addition, extracts of *Aster scaber* and *Artemisia dubia* were also demonstrated to have anti-nosemosis activity and this activity was increased when extracts from both plants were used together (Kim et al., 2016). A study that examined methanolic extracts from leaves used in traditional medicine in Indonesian found that several Indonesian plants were able to inhibit microsporidia, the extract from *Diospyros sumatrana* was shown *in vitro* to inhibit *Enc. cuniculi* and its activity was similar to that of albendazole against *Enc. cuniculi* (Sak et al., 2017). Sulfated polysaccharides extracted from several algae strains significantly reduced the parasite load of *N. ceranae* and improved the survival rate of infected bees (Roussel et al., 2015). Given these observations with various plant extracts that inhibit the proliferation of microsporidia, further work now needs to focus on the purification and structural identification of the key antimicrosporidial compounds in these extracts.

Propolis is a hard resinous hive product collected by honeybee workers from the juices of various plants. The chemical composition of propolis is quite complicated, including aldehydes, polysaccharides, ketones, terpenes, steroids, amino acids, hydrocarbons, and several other compounds (Bankova et al., 1983; Marcucci, 1995). Propolis exhibits anti-bacteria, anti-virus, and anti-fungus activity. Several studies have examined if propolis can control microsporidiosis. Ethanol extracts of propolis obtained from propolis structures of the stingless bee *Trigona apicalis* significantly reduced the *N. ceranae* infection rate and bee mortality rate (Bankova et al., 1983). In addition, this propolis extract treatment increased the trehalose levels and hypopharyngeal gland protein content in treated bees compared to an untreated control group (Suwannapong et al., 2018). Dichloromethane extract of propolis from Upstate New York greatly reduced *N. ceranae* spore loads in a dose-dependent manner (Burnham et al., 2020). A study in Italy that evaluated an ethanol extract of propolis from the honey bee *A. mellifera* demonstrated that treatment decreased the spore load of *N. ceranae*-infected worker bees (Mura et al., 2020). In addition, the food consumption and longevity of the propolis treated group increased. High performance liquid chromatography analysis revealed that there are many organic compounds belonging to flavones, flavonols, and simple phenols in the propolis extract and the active antimicrosporidial compounds were not identified. More research is needed on the application of propolis for the control of microsporidiosis, including the purification and identification of the active compounds in the ethanol extracts as well as studies that examine the activity of these propolis extracts against other microsporidia such as *N. bombycis* and *Encephalitozoon* spp.

Probiotics have been used in both vertebrates and invertebrates to modulate and maintain intestinal health, and they have also been utilized as adjunctive therapy in the treatment of gastrointestinal infections (Silva et al., 2020). *Lactobacillus kunkeei* strains isolated from the gut of bees have shown a potential beneficial effect of decreasing *N. ceranae* infections (Arredondo et al., 2018). *Enterococcus faecium* also significantly reduced *N. ceranae* load in infected bees (Borges et al., 2021). The protective effect of probiotics could be due to metabolites produced by bacteria, such as bacteriocin and surfactin (Porrini et al., 2010; Mossallam et al., 2014). Unfortunately, other studies have demonstrated that probiotics are ineffective for the prevention and treatment of microsporidiosis. Several commercial probiotics and prebiotics have been demonstrated in controlled trials to be ineffective in the treatment and prevention of nosemosis in *N. ceranae* (Ptaszynska et al., 2016). A randomized placebo-controlled study in AIDS patients with non-infectious diarrhea or gastrointestinal symptoms caused by microsporidia indicated that there were no significant differences in patients treated with *Lactobacillus rhamnosus* compared to a placebo treatment group (Salminen et al., 2004). The role of the microbiome in infectious disease therapy and pathogenesis is a rapidly evolving area of investigation. Future research on the isolation, clinical efficacy, and antagonistic mechanisms of various probiotics is clearly needed for the development of this therapeutic approach.

## Conclusion

**FIGURE 2:**
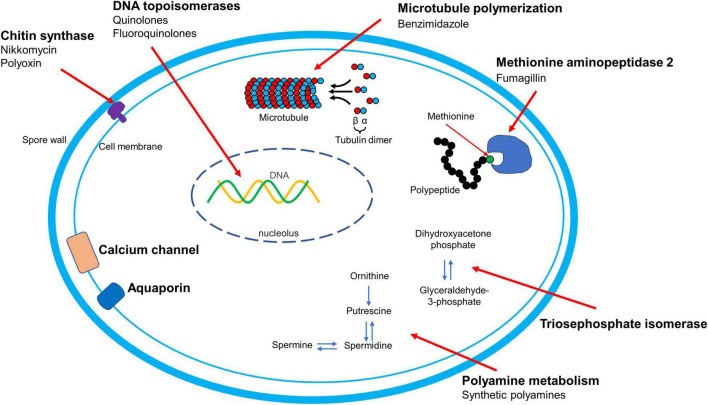
∣ Mechanism of action of therapy and therapeutic targets for microsporidiosis. The names of therapeutic targets are presented in bold. The mode of action of orlistat and nitazoxanide are to be determined and are not shown in this figure.

Microsporidia have become not only important emerging human pathogens in immune competent and immune-compromised individuals, but are also major threats to industries such as sericulture, apiculture, and aquaculture. The known therapeutic targets for microsporidiosis mentioned are summarized in Figure 2. The currently approved drugs for the treatment of microsporidiosis are albendazole and fumagillin, which target β-tubulin and MetAP2, respectively. Fumagillin has a broader antimicrosporidial activity compared to albendazole as it can inhibit *Encephalitozoon* spp., *V. corneae*, and *Ent. bieneusi*, while albendazole has limited activity against *V. corneae* and *Ent. bieneusi*. Apart from these two well studied targets, more therapeutic targets are still required for microsporidiosis drug development. Chitin is a crucial component of the spore wall. Microsporidia have been shown to have a single chitin synthase gene in their genomes, making this an excellent potential therapeutic target as vertebrates do not have this biochemical pathway. Drugs that target the polyamine pathway have been shown to inhibit microsporidia *in vitro* and to be effective in experimental murine microsporidiosis. These polyamine analogues are promising therapeutic agents that deserve further study. Some studies have demonstrated that fluoroquinolones can inhibit microsporidia, however, microsporidia lack type IV topoisomerase which is the known target of fluoroquinolones. Studies on the target of these drugs in microsporidia are clearly needed to further the development of fluoroquinolones as therapeutic agents against microsporidia. The effect of orlistat on microsporidia in experimental murine models is intriguing. This observation suggests that drugs that target lipid metabolism could be useful therapeutic agents for microsporidiosis as well as illustrates that non-absorbed drugs in the gastrointestinal tract can still have activity against microsporidia. Several of the previously mentioned chemicals or their derivatives are already in wide use for other purposes. Investigators are also screening FDA-approved drug libraries to repurpose them for microsporidiosis. This approach holds promise to accelerate development as these repurposed drugs do not require as much of an investment as novel molecules. Complementary and alternative medicine strategies for microsporidiosis need to be carefully evaluated for efficacy as illustrated by controlled studies that demonstrated that several widely used natural treatments for nosematosis were ineffective. Extracts from medicinal plants have been demonstrated to have antimicrosporidial activity and the active component in these extracts need to be identified in order to further develop these therapies. Microsporidia have reduced genomes with corresponding reductions in anabolism and catabolism providing opportunities for the development of agents that target critical enzymes in these streamlined pathways. Microsporidia also have a unique mechanism of invasion requiring spore germination and extrusion of a unique organelle, the polar tube, for successful infection transmission. Screening compound libraries to identify compounds that interfere with this unique invasion process could be a fruitful avenue of investigation for the identification of new therapeutic agents.

## Author Contributions

JW, LW, and ZZ conceptualized and wrote the draft of the manuscript. ZF collected and prepared data about alternative medicine strategies. All the authors revised and approved the final manuscript.

## Conflict of Interest

The authors declare that the research was conducted in the absence of any commercial or financial relationships that could be construed as a potential conflict of interest.

## Publisher’s Note

All claims expressed in this article are solely those of the authors and do not necessarily represent those of their affiliated organizations, or those of the publisher, the editors and the reviewers. Any product that may be evaluated in this article, or claim that may be made by its manufacturer, is not guaranteed or endorsed by the publisher.

## Footnotes

1
http://www.niaid.nih.gov/topics/biodefenserelated/biodefense/pages/cata.aspx

## Abbreviations

*A. algerae*: *Anncaliia algerae*
*A. penaei*: *Agmasoma penaei*
AIDS: acquired immune deficiency syndrome
*A. mellifera*: *Apis mellifera*
*Enc. cuniculi*: *Encephalitozoon cuniculi*
*Enc. hellem*: *Encephalitozoon hellem*
*Enc. intestinalis*: *Encephalitozoon intestinalis*
*Ent. bieneusi*: *Enterocytozoon bieneusi*
EHP: *Enterocytozoon hepatopenaei*
*F. hepatica*: *Fasciola hepatica*
MetAP1: methionine aminopeptidase type 1
MetAP2: methionine aminopeptidase type 2
*N. apis*: *Nosema apis*
*N. bombycis*: *Nosema bombycis*
*N. ceranae*: *Nosema ceranae*
*S. cerevisiae*: *Saccharomyces cerevisiae*
*V. corneae*: *Vittaforma corneae*.
